# A study protocol for a randomised controlled trial to evaluate the effectiveness of a dog-facilitated physical activity minimal intervention on young children’s physical activity, health and development: the PLAYCE PAWS trial

**DOI:** 10.1186/s12889-020-10034-7

**Published:** 2021-01-06

**Authors:** Michelle Ng, Elizabeth Wenden, Leanne Lester, Carri Westgarth, Hayley Christian

**Affiliations:** 1 Telethon Kids Institute, University of Western Australia, Crawley, WA Australia; 2 School of Population and Global Health, University of Western Australia, Perth, WA Australia; 3 School of Human Sciences, University of Western Australia, Crawley, WA Australia; 4 Department of Livestock and One Health, Institute of Infection, Veterinary and Ecological Sciences, University of Liverpool, Leahurst Campus, Neston, UK

**Keywords:** Children, Dog, Dog walking, Dog play, mHealth, Development, Physical activity

## Abstract

**Background:**

Pet ownership brings many health benefits to individuals. In children developmental benefits can extend to improved self-esteem, better social competence and decreased loneliness. The majority of households with children own a dog, however only a small proportion of children gain the benefits of dog ownership through dog walking and play. There are few intervention studies investigating the impact of dog-facilitated physical activity in children. The PLAYCE PAWS study aims to test a minimal-contact intervention through the use of mobile health (“mhealth”) strategies, i.e. text (SMS) messages, to parents to encourage their children to walk and play with their dog more, and evaluate the impact on children’s overall physical activity and development.

**Methods/design:**

The PLAYCE PAWS intervention study will target parents in dog-owning families with children aged 5 to 8 years in Perth, Western Australia. Approximately 150 dog-owning parents and children will be randomly allocated into either one of two intervention groups or a ‘usual care’ control group. The first intervention group will receive SMS messages over 4 weeks to encourage and prompt parents to undertake dog walking and dog play with their child. The second intervention group will receive the same text messages, plus a dog pedometer and personalised ‘dog steps’ diary for their child to complete. Parent-reported outcome measures include changes in children’s dog walking and play, overall physical activity, socio-emotional development, self-regulation, self-esteem, empathy, and level of attachment to their dog.

**Discussion:**

The PLAYCE PAWS study appears to be the first to examine the effectiveness of a low-cost, mhealth intervention for increasing young children’s physical activity through dog walking and play. Given the high prevalence of dogs as family pets, this study presents a valuable opportunity to investigate if mHealth interventions encourage children to walk and play with their dog more, and if there are any associated impact on children’s overall physical activity and socio-emotional well-being. If effective, a larger trial or program could be implemented at low-cost and with wide reach in the community.

**Trial registration:**

ANZCTR, ACTRN12620000288921. Registered 4th March 2020 - Retrospectively registered.

## Background

### Dog ownership and children’s physical activity benefits

Pet ownership is associated with a number of physical, mental and emotional health benefits [1–3]. There is a growing body of research on the physical activity benefits of dog ownership. In adults, dog ownership is associated with higher physical activity levels and increased likelihood of meeting physical activity recommendations [4, 5]. Fewer studies have been conducted on children but overall they show that children from dog-owning families accumulate more physical activity [6–8], and are more likely to meet physical activity recommendations [6, 9]. Furthermore, children who walk their dog, play in the street and yard more and are more independently mobile compared with children who don’t walk their dog [10].

A large proportion of households with children own a dog: in the U.S. and Australia 50–70% of households with children have a dog [8, 11, 12], and in the UK approximately 22–24% of households own a dog [13, 14] with dogs more common in households with children [14]. Despite this high level of dog ownership, many dog-owning children do not engage in any dog-facilitated physical activity, in particular dog walking [6, 7, 15]. An Australian study found only 23% of 5–6 year olds ever walked their dog, walking on average 1.7 times per week with their dog [8]. Increased walking resulting from dog ownership may be an overlooked mechanism for increasing children’s physical activity [6].

### Dog ownership and children’s development

Dog ownership has been associated with other psychological, social and developmental benefits in children. These include better social-emotional development [11], improved self-esteem, autonomy, empathy, trust and self-confidence, increased feelings of safety, social competence [16–18], and family cohesion [19]. For example, a longitudinal study of more than 4000 Australian children aged 5 to 7 years found that dog ownership was associated with lower abnormal scores on any of the Strengths and Difficulties Questionnaire scales (a measure of a child’s social–emotional development) [11]. One of the mechanisms through which the benefits of pets may be conferred to children could be through the level of attachment to a pet [20, 21]. There is some evidence to suggest that children who are more attached to their dogs spend more time being active with them [15, 22].

**Fig. 1:**
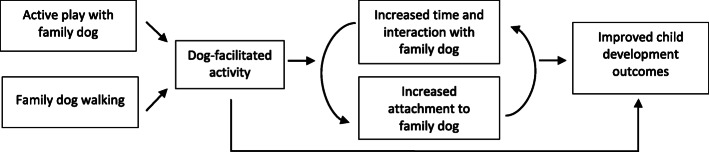
Theoretical model of the relationship between family dog ownership, dog-facilitated and development in young children

Key factors influencing how dog ownership facilitates improved developmental outcomes for children relates to the quality of the child-dog relationship. This includes factors such as the bond and attachment a child has to the family dog [16, 22] as well as the time spent interacting with the family dog. Since dog-facilitated play and family dog walks enables children to spend more time interacting and bonding with their dog, it may be a key mechanism for facilitating increased attachment and developmental benefits such as improved self-esteem, self-regulation and empathy. In addition, regular physical activity has been shown to beneficial for children’s socio-emotional development (such as enhanced social skills and emotional intelligence), mental health (such as reduced depression and anxiety problems) and better sleep [23–25]. Based on key concepts from relationship psychology [26] and attachment theory [27], a theoretical model of the proposed relationships between dog ownership, dog-facilitated physical activity and developmental outcomes in young children is shown in Fig. 1.

### Intervention studies to increase dog-facilitated physical activity

Intervention studies to promote dog-facilitated physical activity have been few, and only one has targeted children. The Children, Parents and Pets Exercising Together (CPET) pilot intervention utilised various strategies (in-person visits, phone calls and text messaging) over 10 weeks to encourage children (*n* = 28, 9–11 year olds) to play/walk their dog [28]. While no significant differences were found between the intervention and control group, mostly due to the small sample size, families found the intervention to be acceptable and feasible.

Two studies in adults have also utilised minimal intervention strategies involving mobile health (mHealth) to increase physical activity through dog walking [29, 30]. A U.S. study (*n* = 102) utilised online social networks to promote weekly neighbourhood dog walks [30] while another (*n* = 105) trialled weekly email messages to promote the benefits and reduce barriers to dog walking [29]. Both studies found mHealth interventions to be effective at increasing dog walking amongst participants in the intervention compared with the control group at follow-up. However, both studies were pilot interventions and thus were limited by small sample sizes.

Physical activity interventions that are relatively low cost, require little face-to-face interaction between participants and those delivering the intervention, and encourage participant involvement in self-selected physical activity may have better long term success [31] compared with traditional interventions which involve closely supervised intensive, and expensive programs [31, 32]. Thus, larger trials with more rigorous research methodology (i.e., random sampling; adjustment for confounders; use of context-specific measures) are needed to determine the best strategies for increasing children’s physical activity through active play and walking with the family dog [16, 33]. In addition, it is important to understand if increased interaction and bonding with the family dog facilitated by increased dog play and family dog walking, leads to other developmental benefits for children.

The aim of this study is to determine if a dog-facilitated physical activity minimal intervention: a) increases the amount of active play, family dog walking and overall physical activity levels; b) increases the amount of time children spend interacting with their family dog and their level of attachment to the dog; and c) improves child development outcomes. We hypothesize that strategies to increase children’s active play and walking with the family dog will improve children’s physical activity levels and development.

## Methods/ design

### Study sample, recruitment, inclusion and exclusion criteria

This is a sub-study of the Play Spaces & Environments for Children’s Physical Activity (PLAYCE) study, which was a cross-sectional observational study conducted from 2015 to 2018 [34]. The PLAYCE study investigated the influence of the home, neighbourhood and early childhood education and care (ECEC) environment on the physical activity levels of children attending ECEC [34]. Participants included 1596 children aged between 2 and 5 years from 104 ECECs in the Perth metropolitan area, Western Australia [35]. Children are now being followed up as they enter full-time school. Specific details of the PLAYCE study’s methods have been published elsewhere [34].

To reach the target sample size (*n* = 150), participants will be recruited from both the PLAYCE cohort [34], and the general community by multiple strategies including advertising in print (newspapers, school and professional association newsletters) and social media (Facebook and Twitter), crowdsourcing (via institutional websites), market research and through snowball sampling. Parents will be invited to participate in the study by filling in an online expression-of-interest form or by contacting the study team directly. The study team will follow-up interested parents to confirm their eligibility to participate. Parents with children between 5 to 8 years and who have a family dog(s) that is well socialised with the child and other people will be eligible to participate. Children with a recognised disability (physical, emotional/behavioural or intellectual) that would affect participation in physical activity will be ineligible.

### Sample size considerations

This project is an exploratory pilot trial, intended in part to inform future sample size calculations. This study aims to recruit 50 participants into each group (total *n* = 150). Based on preliminary analysis of PLAYCE data [35] and the work of Morrison et al. 2013 (CPET study, 2013) [36], this study will have more than 80% power to detect a one-unit difference in the pre-post change in the number of times per week children play or walk with their dog between the intervention and control groups. There are no comparable intervention data in this young group of children to accurately inform a power calculation. However, based on data from the CPET study [36], and the PLAYCE study [35], it is expected that the response within each subject group will be normally distributed with a standard deviation of 1.5. If the true difference in the experimental and control means is one additional dog walk or play session with the dog per week, we will be able to reject the null hypothesis that the population means of the experimental and control groups are equal with a probability (power) of 0.91. The Type I error probability associated with this test of the null hypothesis is 0.05. This study is also sufficiently powered to detect a difference in children’s physical activity, and development outcomes between intervention and control groups, since the outcome measures are context specific (e.g., dog walking/play and home-based outdoor play) compared with the CPET study (overall physical activity) [36].

### Design and randomisation

**Fig. 2:**
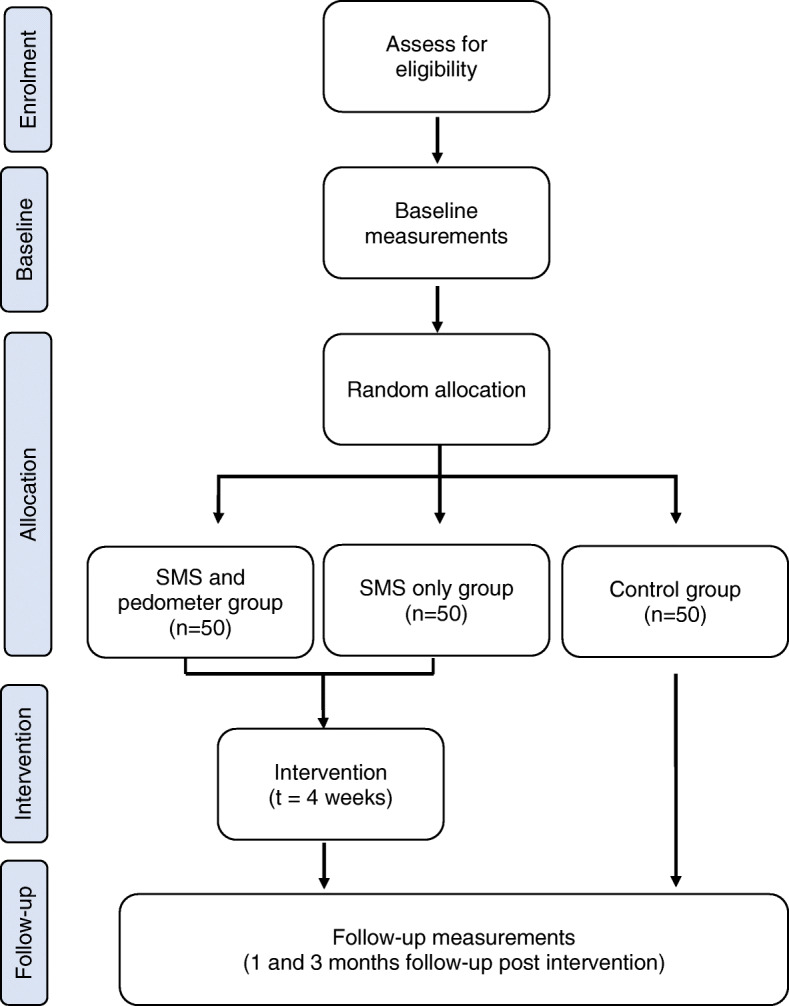
Overview of study design and recruitment process for the PLAYCE PAWS trial

This is a block randomised controlled study with two intervention groups and a ‘usual care’ control group (see Fig. 2) with equal sample sizes in each group (*n* = 50/group; 3 groups; total = 150). Baseline measurements will be collected after eligible participants are screened and recruited (T0). Participants will then be randomly allocated in staggered blocks into one of the three groups. Follow-up measurements will be collected at one-month (F1) and three-months post-intervention (F2) (see Table 1).

**Table 1:** SPIRIT flow diagram of the PLAYCE PAWS trial protocol

Time point	-T1	T0	T1	F1	F2
	Enrolment	Baseline/Allocation	Intervention	Follow-up 1-month	Follow-up 3-months
**Enrolment**					
Eligibility screening	X				
Informed consent	X				
Allocation		X			
**Intervention**					
SMS only intervention group		X	X	X	X
SMS and pedometer intervention group		X	X	X	X
Usual care group (Control)		X		X	X
**Assessment (parent report)**^**a**^					
*Physical activity variables*					
Structured physical activity		X		X	X
Unstructured physical activity (including dog play and family dog walking)		X		X	X
Outdoor play time		X		X	X
*Development variables*					
Social-emotional development		X		X	X
Self-esteem		X		X	X
Empathy		X		X	X
Self-regulation		X		X	X
*Dog attachment variables*		X		X	X
*Socio-demographic information*		X		X	X

^a^See ‘Methods: Outcome measures’ section below for full details of variables.

### Intervention and control groups

Physical activity -based minimal intervention strategies will be tested over a four-week period, consisting of mHealth SMS message prompts to parents and a dog pedometer and personalised dog steps diary for children. The study consists of two intervention groups: the ‘SMS only group’ and ‘SMS and dog pedometer group’ and a ‘usual care’ control group. Both intervention groups will be sent a personalised mobile phone SMS message three times a week (two sent during the work week and one sent on the weekend) to motivate and encourage parents to support their child to either walk and/or play with their dog each day. Examples include: “Has <dog> been outdoors today? Spring is a great time to go for a walk with <dog> and <child>“; “Physical activity can make us feel cheerful. What activity will you, <child> and <dog> do today?”. The ‘SMS and dog pedometer’ group will also receive a Yamax SW200 pedometer for their dog’s collar and a personalised dog steps diary children can use to record the number of pedometer steps their dog does during play or walking. We hypothesize that the use of the dog pedometer and personalised dog steps diary will provide additional encouragement and motivation for parents to take their child and dog for a walk or play each day, and may result in a larger effect compared with the SMS only intervention group and control group.

In order to help facilitate dog walking and dog play, participants in both intervention groups will be provided with information about dog friendly parks, trails and beaches; games for children to play with their dog; and tips about how children can safely interact with their dog. Participants in the control group will be asked to continue their normal routine without any contact from researchers. To ensure fair access to any beneficial outcomes of the project, the control group will receive the same resources as the intervention groups at the end of the study (i.e., information about dog friendly parks, trails and beaches; games for children to play with their dog; and tips about how children can safely interact with their dog).

### Outcome measures

Parents will complete three online surveys to measure changes in children’s dog play, family dog walking, overall physical activity, self-regulation, self-esteem, empathy, and level of attachment to their dog.

#### Change in children’s physical activity

Existing items from the PLAYCE parent-report survey [34] and adapted from the Healthy Active Preschool Years Study (HAPPY Study) [37] will be used to measure children’s frequency of active play with their dog, family dog walking, structured (e.g., swimming, gymnastics, football) and unstructured (e.g., playing in the yard, riding bike, active play with toys) physical activity (response scale for all items: ‘never/rarely’, less than once/week, 3–4 times/week, 5–6 times /week, daily). The reliability of these items is sound (e.g., unstructured physical activity items intraclass correlation (ICC) = 0.63; structured physical activity items ICC = 0.70) [37]. Outdoor play time will be measured using an established tool [38] where parents report the amount of time their child spends outdoors (0, 1–15, 16–30, 31–60, >60mins) across three periods of the day (morning to before lunch; after lunch to 6 pm; 6 pm to bedtime). These items have previously been validated against young children’s accelerometer data (*r* = 0.33, *p* < 0.001) [38].

#### Change in children’s development

*Social-emotional development* will be measured using the parent-report Strengths and Difficulties Questionnaire (SDQ) [39]. The SDQ is a validated and commonly used 25-item (scale: ‘not true’, ‘somewhat true’, ‘certainly true’) instrument that measures the social and emotional well-being of children aged 2–17 years. Items are combined to create five sub-scales (emotional symptoms, conduct problems, hyperactivity-inattention, peer relationship problems, pro-social behaviour) and a total difficulties score [39]. The parent-report version of the SDQ has satisfactory reliability [40].

*Self-esteem* will be measured using the Rosenberg Self-Esteem Scale [41]. This instrument includes ten items (5 point scale: strongly disagree to strongly agree) measuring both positive and negative feelings that a child has [41]. The scale has good reliability; test-retest correlations 0.82–0.88, and Cronbach’s alpha for various samples range 0.77–0.88 [42, 43].

*Empathy* will be measured using the Young Children’s Empathy Measure [44]. Parents report their child’s ability to identify sadness, fear, anger and happiness in four scenarios: sadness (a child has just lost its best friend), fear (a child is chased by a big, nasty monster), anger (a child really wants to go out but is not allowed) and happiness (a child is going to its most favorite park to play’. The response scale options are ‘exact match to the intended emotion’; ‘similar emotion’; ‘some emotion’; ‘non-emotional response’; ‘no response’. The measure has acceptable internal reliability (Cronbach alpha 0.69) [44]. To measure the child’s empathy towards dogs, the questions will be repeated with ‘dog’ as the subject of the four statements [44].

*Self-regulation* will be measured using the Modified Child Problem Behaviour Checklist from the Fast Track Project [45]. This checklist has been generated using items from the Child Behaviour Checklist [46], the Revised Behaviour Problem Checklist [47] and other items developed by the program’s investigators (for further details, see Lochman & The Conduct Problem Prevention Research Group [48]). The checklist includes 20 items measuring the frequency of child externalizing behavior problems (‘none of the time, ‘some of the time’, ‘most of the time’, ‘all of the time’), and has demonstrated high internal consistency (α = 0.87) [48].

The level of *attachment the child has to their family dog* will be measured using items from the Dogs and Physical Activity Tool (DAPA Tool) [49]. This Tool has been shown to be a reliable tool for measuring adult-reported levels of attachment to their dogs, with good to excellent ICC values (ICC = 0.65–0.92) [49]. The Inclusion of Other in the Self (IOS) Scale by Aron and colleagues [50], which measures how close a respondent feels with another person will also be used and has been previously modified to measure how close the child feels about ‘dog’ instead of ‘another person or group’. [51, 52].

#### Potential confounders

Potential confounders will include parent and child socio-demographic factors (e.g., age, gender, ethnicity, socio-economic status [53]), child’s sleep [53] and preference for physical activity [54]. We will adjust for baseline levels of children’s dog play and family dog walking. To account for any seasonal variations in children’s physical activity-related variables we will also collect information on weather conditions from the Australian Bureau of Meteorology [55].

### Participant response and retention

In order to ensure optimum response rates a number of response and retention methods will be employed. A SMS reminder message will be sent to participants 1 week before the survey is due for completion, followed by an email containing a link to the online survey the week the survey is due for completion. A reminder email will be sent to participants who have not completed the survey 1 week later. If further prompting to complete the survey is required, a follow-up phone call and /or SMS message reminder, as well as a paper copy of the survey, will be posted to participants with a reply-paid envelope after 2 weeks. To encourage surveys to be completed in a timely manner, participants who complete the survey within a four-week period will be eligible to go into the draw to win a $100 AUD gift card. Incentives, including packets of dog treats and a frisbee, will be given at different stages of the study (e.g., completion of first and final follow-up surveys) to encourage the timely completion and return of study materials and equipment (pedometers and diaries). At the end of the study, all participants will be provided with a report of the study key findings.

### Statistical analysis

Data will be entered electronically on a secure file storage system and password protected. Data will be anonymised by assigning a unique identification number to each participant.

Analyses will involve repeated measures linear models adjusting for relevant confounding variables. The dependent variables will be mean change in 1) frequency of dog play and walking the dog, 2) overall physical activity levels, and 3) development (social-emotional development as measured by the SDQ, self-esteem, empathy, self-regulation, child-dog attachment scores) between pre- and post-intervention time points. The independent variable will be the group (intervention group 1, intervention group 2 or control) participants are assigned to. Between group differences in baseline variables will be examined. Analyses will be based on intention to treat. Effect modifiers and confounders such as baseline levels of dog play, dog walking frequency and socio-demographic factors will be examined and adjusted for where necessary.

### Dissemination of project findings

A study report presenting overall findings of the research will be prepared for all participants and stakeholders. Peer-reviewed publications will allow the results to be disseminated to the scientific community.

## Discussion

The aim of the current study is to test if a dog-facilitated physical activity minimal intervention increases the amount of time young children spend interacting and bonding with their dog, thereby improving physical activity levels and developmental outcomes.

Of the studies examining the relationship between dog walking and physical activity in children and adolescents [6, 7, 15, 22, 56–59], only one has been conducted with younger children (less than 9 years old) [8]. Most studies have not considered the types of dog-facilitated physical activity young children are likely to engage in (e.g. play with family dog, family dog walking) [7]. Importantly, there is a lack of intervention studies to determine if dog-facilitated physical activity can increase young children’s overall physical activity levels. Our study aims to address this evidence gap.

The current study also seeks to investigate if children who spend more time engaged in play and walking with the family dog improve their bond and attachment with their dog, which may directly or indirectly help facilitate aspects of a child’s development. There is some evidence suggesting that children are more likely to report walking their dog if they have a high attachment to it [60]. There is also recent strong evidence that dog ownership (and dog-facilitated physical activity) is associated with improved social-emotional development in young children. For example, a recent Australian study of 1646 children aged 2 to 5 years found that dog ownership was associated with reduced likelihood of conduct problems, peer problems, and increased likelihood of prosocial behaviour compared with children without a dog [61]. Another small study of 27 8–12 year olds found children’s behaviour improved (less naughty, more cooperative) 1 month after acquiring a dog compared to non-dog owners, however no significant differences were observed at the 6- and 12- month follow-up [62]. Thus, there is a need for larger intervention studies to investigate whether increasing dog facilitated play and walking has a positive impact on young children’s development.

Many traditional physical activity interventions tend to be labour intensive, costly, with short term effects lost once support has been withdrawn [63]. It has been suggested that a ‘paradigm shift’ is required in physical activity interventions, moving to encouraging more ‘purposeful’ activity such as walking the dog or walking or biking to destinations [63]. In addition, even the provision of single contact education (e.g. providing education material outlining the benefits of dog walking and dog walking tips) of the importance of dog walking has been shown to be beneficial in increasing adult physical activity levels [64]. Past adult dog walking intervention studies have shown promise but lack evidence of their ability for scale up and application to other population groups such as children [33].

This study utilises simple mHealth strategies which can be easily scaled up and incorporated into mass media campaigns targeting population-level walking across diverse community settings. If shown to be effective, the PLAYCE PAWS intervention may provide an opportunistic, low cost, wide reaching strategy for increasing children’s physical activity and improving developmental outcomes.

## Data Availability

The datasets used and/or to be analysed during the current study, in addition to data collection forms are available from the corresponding author on reasonable request.

## Abbreviations

mHealth: Mobile health
PLAYCE: Play Spaces & Environments for Children’s Physical Activity
SDQ: Strengths and Difficulties Questionnaire
SMS messages: Text messages
SPIRIT: Standard Protocol Items: Recommendations for Interventional Trials
